# Epigenetic silencing of miR-483-3p promotes acquired gefitinib resistance and EMT in EGFR-mutant NSCLC by targeting integrin β3

**DOI:** 10.1038/s41388-018-0276-2

**Published:** 2018-05-02

**Authors:** Jinnan Yue, Dacheng Lv, Caiyun Wang, Ling Li, Qingnan Zhao, Hongzhuan Chen, Lu Xu

**Affiliations:** Department of Pharmacology and Chemical Biology, Shanghai Jiao Tong University School of Medicine, Shanghai, China

## Abstract

All lung cancers patients with epidermal growth factor receptor (EGFR) mutation inevitably develop acquired resistance to EGFR tyrosine kinase inhibitors (TKI). In up to 30% of cases, the mechanism underlying acquired resistance remains unknown. MicroRNAs (miRNAs) is a group of small non-coding RNAs commonly dysregulated in human cancers and have been implicated in therapy resistance. The aim of this study was to understand the roles of novel miRNAs in acquired EGFR TKI resistance in EGFR-mutant non-small cell lung cancer (NSCLC). Here, we reported the evidence of miR-483-3p silencing and epithelial-to-mesenchymal transition (EMT) phenotype in both in vitro and in vivo EGFR-mutant NSCLC models with acquired resistance to gefitinib. In those tumor models, forced expression of miR-483-3p efficiently increased sensitivity of gefitinib-resistant lung cancer cells to gefitinib by inhibiting proliferation and promoting apoptosis. Moreover, miR-483-3p reversed EMT and inhibited migration, invasion, and metastasis of gefitinib-resistant lung cancer cells. Mechanistically, miR-483-3p directly targeted integrin β3, and thus repressed downstream FAK/Erk signaling pathway. Furthermore, the silencing of miR-483-3p in gefitinib-resistant lung cancer cells was due to hypermethylation of its own promoter. Taken together, our data identify miR-483-3p as a promising target for combination therapy to overcome acquired EGFR TKI resistance in EGFR-mutant NSCLC.

## INTRODUCTION

EGFR tyrosine kinase inhibitors (TKI) including gefitinib and erlotinib have demonstrated dramatic efficacy in non-small cell lung cancer (NSCLC) patients with EGFR-activating mutation [1]. In general, activating EGFR mutations are more commonly observed in non-smoking, female, Asian patients with adenocarcinoma histology, which is one of the most common histological subtypes of NSCLC. Despite impressive initial response, almost all patients eventually have a relapse due to the occurrence of acquired resistance. Several mechanisms leading to acquired resistance have been demonstrated, including EGFR T790M mutation, MET amplification, PIK3CA mutation, AXL activation, small cell lung cancer (SCLC) transformation, or acquiring an epithelial-to-mesenchymal transition (EMT) phenotype [2–7]. To note, these mechanisms of acquired resistance can take place together in different subclones of the same tumor at the same time. However, the mechanisms remain unknown in ~ 30% of cases.

MicroRNAs (miRNA) are a class of small non-coding, endogenous RNAs of 21–25 nucleotides in length, which repress target genes expression by directly binding to the 3′-untranslated region (UTR) of target gene mRNAs and promoting degradation or repressing translation of these mRNAs. Deregulated miRNA expression has been associated with tumorigenesis, cancer progression, and response to therapy [8–10]. Modulating miRNA expression in cancers by targeted delivery of miRNA inhibitors or mimics appears to be a promising strategy for cancer therapy. Several miRNA therapeutics are already in clinical trial stage. For example, MRX34, a liposome-formulated mimic of miR-34a, which is often downregulated in human malignancies and functions as tumor suppressor, has entered into phase I clinical trials (NCT01829971). Recently, the involvement of miRNA in acquired resistance to EGFR TKI has been reported. For example, miR-21 has been reported to mediate acquired EGFR TKI resistance by targeting phosphatase and tensin homolog (PTEN) [11, 12]. In addition, combination therapy of EGFR TKI and miRNA mimics or inhibitors has shown to have a synergistic effect in inhibiting NSCLC cell growth [13]. Thus, it seems that miRNAs may represent promising candidates for adjuvant therapy for NSCLC patients who develop resistance to long-term EGFR TKI treatment. However, our knowledge of how miRNAs modulate tumor initiation, development, and progression, especially how they affect treatment response is not adequate.

The aim of our investigation was to identify novel miRNAs contributed to EGFR TKI acquired resistance in NSCLC. Our study was the first one to identify that miR-483-3p, a miRNA highly conserved among placental mammals, was significantly silenced in gefitinib-resistant NSCLC cells and lung tissues. miR-483-3p has been reported dysregulated in some types of tumors [14–23]. But the roles of miR-483-3p in NSCLC were largely unknown. Herein, functional studies demonstrated that miR-483-3p increased sensitivity of gefitinib-resistant NSCLC to gefitinib by inhibiting resistant cell proliferation and promoting apoptosis. Moreover, miR-483-3p inhibited EMT phenotype and inhibited migration, invasion, and metastasis in gefitinib-resistant NSCLC cells. Furthermore, mechanistic studies demonstrated a mechanism by which downregulation of miR-483-3p activated FAK/Erk pathway via upregulating integrin β3. The miR-483-3p silencing in gefitinib-resistance cells was due to hypermethylation of its promoter. Together, these studies identify a miR-483-3p/integrin β3/FAK/Erk axis as a new mechanism and target for EGFR TKI acquired resistance in NSCLC.

## Results

### The expression of miR-483-3p is dramatically decreased in gefitinib-resistant NSCLC in vitro and in vivo

**Fig. 1:**
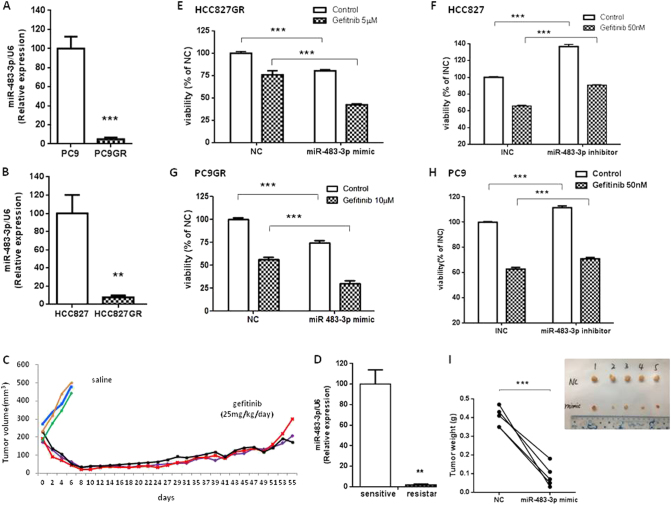
miR-483-3p regulates gefitinib sensitivity in EGFR-mutant lung cancer in vitro and in vivo. **a**, **b** Quantitative RT-PCR analysis of hsa-miR-483-3p levels in PC9GR compared with PC9 **a** and in HCC827GR compared with HCC827 **b**. RNU6B is used for normalization. **c**, **d** HCC827 xenograft tumors in immunocompromised mice were treated with gefitinib (25 mg/kg/day) orally until tumor regrew or treated with saline for 1 week. **c** The growth curve of HCC827 xenograft tumors. **d** Quantitative RT-PCR analysis of miR-483-3p levels in regrown HCC827 xenograft tumors treated with gefitinib (resistant) for 6–8 weeks relative to saline-treated control tumors (sensitive). **e**–**h** Cell viability by CCK8 assay of indicated cells transiently transfected with miR-483-3p mimic, negative control (NC), miR-483-3p inhibitor, or inhibitor negative control (INC) as indicated in the presence or absence of indicated concentration of gefitinib for 72 h. **i** Agomir-483-3p mimic or agomir-NC was intratumorally injected into left or right regrown/resistant HCC827 xenograft tumor of each mouse, respectively, once every 4 days for a total four injections. Tumors were weighted and photographed (*n* = 5). For all panels: *n* = 5. ** *P* < 0.01; *** *P* < 0.001

To identify novel miRNAs contributed to acquired resistance, first, we generated in vitro model by growing gefitinib-sensitive NSCLC cell lines (HCC827, PC9) in gefitinib with escalating concentrations as we previously reported [24]. Gefitinib-resistant sublines were highly resistant to gefitinib and either T790M or MET amplification was not detected in these resistant cell lines, which was consistent with other studies [11, 12, 25–27]. We then performed genome-wide miRNA chip analysis in gefitinib-sensitive parent cell lines and gefitinib-resistant cell lines using miRNA microarrays (GSE110815). A total of 15 miRNAs were detected to be increased, whereas 11 miRNAs were decreased (fold change ≥2) (Fig. S1A). Our focus was on miR-483-3p, as it was one of the most downregulated miRNAs in both GR cells. To validate the microarray data, quantitative real-time polymerase chain reaction (qRT-PCR) was performed and confirmed that miR-483-3p was dramatically decreased in both GR cells (Fig. 1a, b). Next, to establish in vivo models, nude mice with subcutaneous HCC827 xenograft tumors (two tumors left and right per mouse) were given saline for 1 week (sensitive) or gefitinib orally over 2–3 months to derive gefitinib-resistant tumors as previously reported [9, 28–30]. Daily gefitinib treatment of HCC827 xenografts led to an initial remarkable tumor shrink and later tumor regrowth in 6–8 weeks (Fig. 1c). In agreement with previous studies [9, 28], neither T790M nor MET amplification was found in these gefitinib-resistant tumors (data not shown). We then examined miR-483-3p expression by qRT-PCR and shown that the expression of miR-483-3p was also dramatically decreased in regrown/resistant tumors as to primary/sensitive tumors from saline-treated littermate control mice (Fig. 1d). Collectively, miR-483-3p was dramatically decreased in gefitinib-resistant NSCLC in vitro and in vivo, suggesting that it could have a role in the regulation of acquired gefitinib resistance. To expand our observations to irreversible EGFR TKI, we generated afatinib-resistant (AR) EGFR-mutant NSCLC cell lines and examined miR-483-3p by RT-qPCR. As expected, miR-483-3p was also dramatically decreased in both AR cell lines (Fig. S1B).

### miR-483-3p re-sensitizes gefitinib-resistant NSCLC to gefitinib in vitro and in vivo

To examine whether downregulation of miR-483-3p associated with acquired resistance to gefitinib in vitro, we modulated miR-483-3p expression by transiently transfecting miR-483-3p mimic or inhibitor into NSCLC cells and then assessed the sensitivity to gefitinib by cell viability. First, we determined the efficacy of transient transfection by examining miR-483-3p in transfected cells by qRT-PCR. As shown in Fig. S2A and S2B, cells transfected with mimic or inhibitor showed a 25–30 fold increase or a 99 % decrease in miR-483-3p level compared with their respective negative controls (NC or INC). Next, CCK8 assay demonstrated that miR-483-3p mimic dramatically decreased the viability of GR cells, whereas miR-483-3p inhibitor slightly increased the viability of parental cells in both presence and absence of gefitinib, as shown in Fig. 1e–h, suggesting that miR-483-3p silencing contributed to acquired resistance and miR-483-3p replenishment increased gefitinib sensitivity in vitro. Furthermore, this notion was investigated using in vivo acquired resistance model as discussed before. Agomir-483-3p mimic and agomir-NC was injected intratumorally once every 4 days for a total of four injections into right and left regrown HCC827-derived xenograft tumor of same tumor-bearing mouse, respectively, which was on daily gefitinib up to sacrifice. After sacrifice, first, the miR-483-3p level in xenograft tumors was examined by RT-qPCR to determine the efficacy of intratumoral injection of agomir-483-3p. It turned out that miR-483-3p was dramatically upregulated in regrown tumors treated with mimic compared with those treated with NC (Fig. S2C). More importantly, as shown in Fig. 1i, intratumoral delivery of mimic dramatically reduced tumor size and weight compared with intratumoral delivery of NC, recapitulating our findings in vitro. Taken together, the data demonstrated that miR-483-3p increased gefitinib sensitivity of gefitinib-resistant NSCLC.

### miR-483-3p inhibits proliferation and promotes apoptosis in vitro and in vivo

**Fig. 2:**
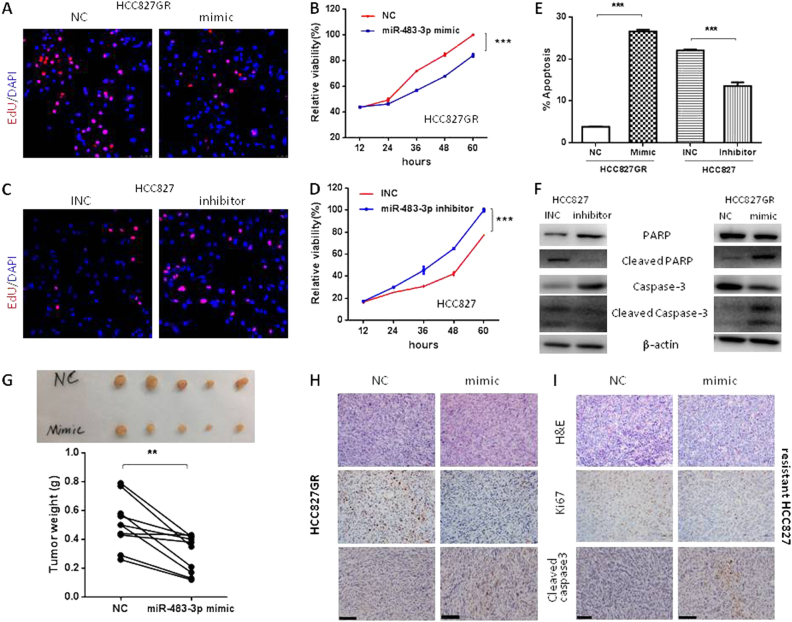
miR-483-3p inhibits growth and promotes apoptosis in gefitinib-resistant cells. **a**–**f** HCC827GR and HCC827 were transiently transfected with miR-483-3p mimic (mimic), negative control (NC), miR-483-3p inhibitor (inhibitor), or inhibitor negative control (INC) as indicated. Cell proliferation was measured by EdU incorporation assay **a**, **c** (red) and cell viability assay **b**, **d**. Cell apoptosis was measured by flow cytometric apoptosis assay **e** and Western blot analysis of PARP and caspase-3 activation **f**. **g**, **h** Xenograft tumors were established using HCC827GR. Agomir-483-3p mimic or agomir-NC was intratumorally injected into left or right HCC827GR-xenograft tumor of each mouse, respectively, once every 4 days for a total four injections. Tumors were weighted and photographed **g** and then sectioned for H & E staining **h**, top panel, Ki67 immunostaining **h**, middle panel, and cleaved caspase-3 **h**, bottom panel (*n* = 5). **i** Agomir-483-3p mimic or agomir-NC was intratumorally injected into left or right regrown/resistant HCC827 xenograft tumor (resistant HCC827) of each mouse, respectively, once every 4 days for a total four injections. Tumors were sectioned for H&E staining **h**, top panel, Ki67 immunostaining **h**, middle panel, and cleaved caspase-3 **h**, bottom panel. For all panels: *n* = 5.** *P* < 0.01; *** *P* < 0.001

To determine whether miR-483-3p inhibits proliferation and/or promotes apoptosis, we modulated miR-483-3p levels in cells and then assessed the effects on proliferation using EdU incorporation and cell viability assays, and apoptosis using flow cytometry and Western blot analysis of apoptosis markers (Caspase-3 and PARP). First, we found that miR-483-3p mimic decreased HCC827GR proliferation (Fig. 2a, b and S3A), whereas miR-483-3p inhibitor increased HCC827 proliferation (Fig. 2c, d and S3B). We also found that miR-483-3p mimics promoted HCC827GR apoptosis, whereas miR-483-3p inhibitor inhibited HCC827 apoptosis (Fig. 2e, f). Next, extending our findings to in vivo, we injected HCC827GR cells subcutaneously into left and right flanks of each nude mouse. And when tumor cells formed solid, palpable tumor with an average volume of 200–250 mm^3^, agomir-483-3p mimic, or agomir-NC was given intratumorally once every 4 days for a total of four injections into left or right tumor of each mouse, respectively. After sacrifice, first, miR-483-3p in xenograft tumors was examined by RT-qPCR to determine the efficacy of intratumoral injection of agomir-483-3p. It turned out that miR-483-3p was dramatically upregulated in HCC827GR-xenograft tumors injected with mimic compared with those with NC (Fig. S2D). Moreover, as shown in Fig. 2g, intratumoral injection of miR-483-3p dramatically reduced tumor size and weight, confirming that miR-483-3p also inhibited resistant tumors growth in vivo. Moreover, IHC analysis verified that miR-483-3p mimic treatment dramatically decreased Ki67 expression and increased cleaved caspase-3 expression in both HCC827GR-xenograft tumors (Fig. 2h) and regrown/resistant HCC827 xenograft tumors (Fig. 2i). Put together, our results established that miR-483-3p inhibited proliferation and promoted apoptosis of gefitinib-resistant lung cancer in vitro and in vivo.

### miR-483-3p inhibits EMT phenotype

**Fig. 3:**
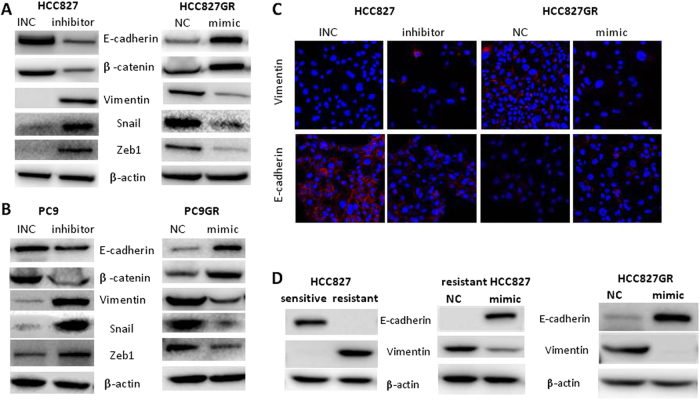
miR-483-3p reverses EMT in gefitinib-resistant cells. **a**–**c** Indicated cells were transiently transfected with miR-483-3p mimic (mimic), negative control (NC), miR-483-3p inhibitor (inhibitor), or inhibitor negative control (INC) as indicated. **a**, **b** Western blot analysis of the expression of EMT markers. **c** Immunostaining analysis of vimentin (red, top panel) and E-cadherin (red, bottom panel). **d** Western blot analysis of E-cadherin and vimentin in HCC827 xenograft tumors treated with gefitinib daily for 6–8 weeks until tumors regrown (resistant) or saline for 1 week (sensitive) (left panel). Western blot analysis of E-cadherin and vimentin in regrown HCC827 xenograft tumors (resistant HCC827) (middle panel) or HCC827GR-xenograft tumors (right panel) intratumorally injected with agomir-483-3p mimic (mimic) or agomir-NC (NC)

EMT phenotype has been connected to acquired EGFR TKI resistance in lung cancer patients [3, 6]. Similarly, EGFR-mutant NSCLC cells may undergo an EMT phenotype during chronic EGFR TKI treatment in vitro as we and others previously reported [7, 24, 31, 32]. To investigate the connection of miR-483-3p in EMT linked to gefitinib resistance, we first modulated miR-483-3p expression in NSCLC cells and assessed the effects on the expression levels of EMT markers using Western blot and immunofluorescence. As we can see in Fig. 3a–c, forced expression of miR-483-3p in GR cells inhibited EMT phenotype, whereas suppression of endogenous miR-483-3p expression in parental cells promoted EMT. Furthermore, consistent with previous study [7], we found that E-cadherin was decreased, whereas vimentin was increased in regrown/resistant HCC827 xenograft tumors compared with primary/sensitive HCC827 xenograft tumors, suggesting EMT phenotype was acquired in gefitinib-resistant tumors (Fig. 3d, left). EMT was reversed in those resistant tumors injected with mimic compared with NC (Fig. 3d, middle). In addition, in HCC827GR-derived xenograft tumors, miR-483-3p mimic also reversed EMT compared with NC (Fig. 3d, right). Taken together, these results demonstrated that in gefitinib-resistant NSCLC, miR-438-3p reversed EMT phenotype, which attributed to increased sensitivity to gefitinib.

### miR-483-3p inhibits migration and invasion in vitro and metastasis in vivo

**Fig. 4:**
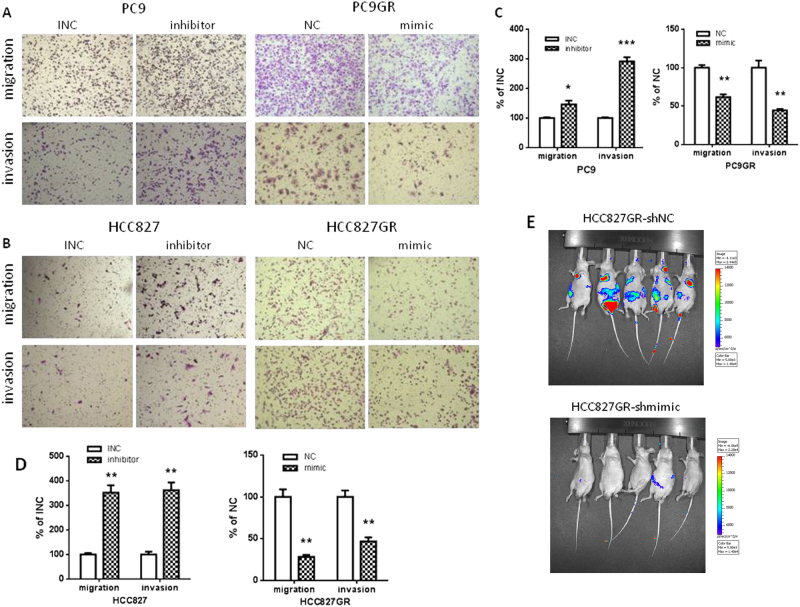
miR-483-3p inhibits migration and invasion in vitro and metastasis in vivo of gefitinib-resistant cells. **a**, **b** Transwell migration and invasion assays of indicated cells transiently transfected with miR-483-3p mimic (mimic), negative control (NC), miR-483-3p inhibitor (inhibitor) or inhibitor negative control (INC) as indicated. **c**, **d** Quantification of **a**, **b**. The number of transwelled cells was counted from at least four independent microscopic fields. For all panels: *n* = 5. * *P* < 0.05; ** *P* < 0.01; *** *P* < 0.001. **e** Bioluminescence imaging of metastasis in immunocompromised mice intravenously injected with luciferase-labeled HCC827GR stably transfected with miR-483-3p mimic (shmimic) or negative control (shNC)

Induction of EMT has been shown to endow cancer cells with improved invasive and metastatic abilities during progression [33]. Next, we examined the roles of miR-483-3p in migration and invasion in vitro, measured by transwell assays. As shown in Fig. 4a–d, in GR cells, miR-483-3p mimic decreased migration and invasion, whereas miR-483-3p inhibitor increased migration and invasion in parental cells. Furthermore, we studied the effects of miR-483-3p on metastasis in vivo. HCC827GR cells were labeled with luciferase and then stably transfected with miR-483-3p mimic (shmimic) and NC (shNC). The efficacy of stable transfection was determined by examining miR-483-3p level by qRT-PCR. As shown in Fig. S2E, cells stably transfected with shmimic showed a 25–30 fold increase in miR-483-3p level compared with shNC. HCC827GR-shmimic and HCC827GR-shNC cells were then injected intravenously via tail vein into nude mice. Metastasis was assessed by whole animal bioluminescence imaging. As shown in Fig. 4e, miR-483-3p overexpression strongly and significantly suppressed metastasis. Collectively, these results demonstrated that miR-483-3p suppressed lung cancer invasion and metastasis.

### Integrin β3 is the direct and functional target of miR-483-3p

**Fig. 5:**
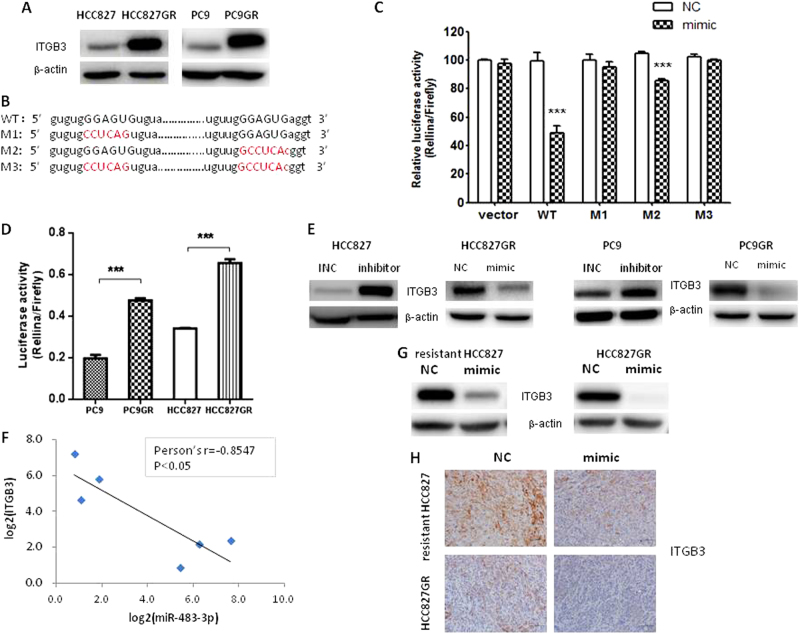
Integrin β3 is a direct target of miR-483-3p. **a** Western blot analysis of integrin β3 expression in indicated cells. **b** The sequences of wild-type (WT) and site-directed mutation (M1-M3) of two predicted binding sites (black capital letter) of miR-483-3p within 3′-UTR of ITGB3 mRNA. **c** Luciferase activity of HEK293 cells co-transfected with indicated vector and miR-483-3p mimic (mimic) or negative control (NC). **d** Luciferase activity in indicated cells transfected with wild-type 3′-UTR of ITGB3 mRNA. **e** Western blot analysis of integrin β3 expression of indicated cells transiently transfected with miR-483-3p mimic (mimic), negative control (NC), miR-483-3p inhibitor (inhibitor), or inhibitor negative control (INC) as indicated. **f** Negative correlation of miR-483-3p and integrin β3 expression in 6 NSCLC cell lines. The levels of miR-483-3p were determined by qRT-PCR and normalized to RNU6B, whereas the levels of integrin β3 were determined by Western blot and normalized to β-actin. *P* and *r* values were calculated using a Spearman correlation test. **g** Western blot analysis of integrin β3 expression of regrown/resistant HCC827 xenograft tumors (resistant HCC827) (left panel) or HCC827GRxenograft tumors (right panel) intratumorally injected with agomir-483-3p mimic (mimic) or agomir-NC (NC). **h** Immunostaining analysis of integrin β3 in sectioned xenograft tumors of **g**. For all panels: *n* = 5. *** *P* < 0.001

To investigate the mechanisms by which miR-483-3p exerted regulatory function on gefitinib-resistant NSCLC, we identified prospective targets of miR-483-3p. Genes contained miR-483-3p binding site(s) in their 3′-UTR, as predicted by miRWalk (http://www.umm.uni-heidelberg.de/apps/zmf/mirwalk/) containing 10 prediction tools were selected and validated by RT-qPCR. Interestingly, ITGB3 gene encoding integrin β3 was one of potential targets of miR-483-3p. Indeed, Western blot analysis showed that integrin β3 was upregulated in resistant cells as to sensitive/parental cells (Fig. 5a). To determine whether ITGB3 is the functional target of miR-483-3p, first we performed 3′-UTR luciferase reporter assays using psiCHECK-2 containing wild-type or mutated ITGB3 3′-UTR fragments. The 3′-UTR of ITGB3 has two predicted miR-483-3p binding sites (Fig. 5b, black capital letter). We mutated these two binding sites individually (M1 and M2) as well as combined (M3) (Fig. 5b, red capital letter). The mutants contained several nucleotide substitutions in the predicted binding site, which should disrupt miRNA binding. miR-483-3p mimic decreased the luciferase activity of ITGB3 3′-UTR wild-type construct (WT) in HEK293 cells by up to 55% (Fig. 5c). The suppression of luciferase activity by miR-483-3p was reduced of ITGB3 3′-UTR M2 compared with wild-type construct and completely abolished of ITGB3 3′-UTR M1 and M3, suggesting that miR-483-3p mainly binds directly to site 1 of the 3′-UTR of ITGB3 (Fig. 5c). In addition, luciferase activity of GR cells transfected with ITGB3 3′-UTR wild-type construct was significantly upregulated than that of parental cells (Fig. 5d), due to dramatically decreased miR-483-3p in GR cells. Next, to determine whether miR-483-3p affects endogenous ITGB3 expression, we modulated the miR-483-3p levels by transiently transfecting GR and parental cells with miR-483-3p mimic and inhibitor, respectively. As demonstrated in Fig. 5e, integrin β3 protein expression level was inversely regulated by miR-483-3p. And at last, an inverse correlation between the expression levels of miR-483-3p and integrin β3 protein was confirmed in 6 NSCLC cell lines (HCC827, PC9, H1975, H292, A549, H1299) (Fig. 5f). To extend the findings to in vivo, we examined the expression of integrin β3 in our in vivo models. First, miR-483-3p mimic downregulated integrin β3 expression level in regrown/resistant HCC827 xenograft tumors (Fig. 5g, left and 5h, upper). In addition, in HCC827GR-derived xenograft tumors, miR-483-3p mimic also downregulated integrin β3 (Fig. 5g, right and 5h, lower). Taken together, these data suggested that miR-483-3p can downregulate integrin β3 level by targeting its 3′-UTR directly.

**Fig. 6:**
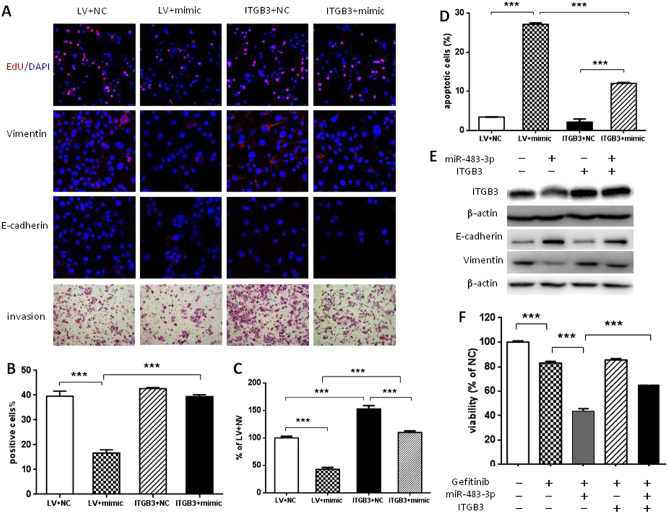
Integrin β3 overexpression rescues miR-483-3p-induced phenotypes. HCC827GR cells were co-transfected with miR-483-3p mimic (mimic) or negative control (NC) and LV-ITGB3 (ITGB3) or LV-GPF (LV) as indicated. **a** EdU incorporation assay (red, upper panel), immunostaining analysis of E-cadherin and vimentin (red, middle two panels), transwell invasion assay (bottom panel). **b**, **c** Quantification of EdU incorporation assay **b** and transwell invasion assay **c** of **a**. **d** Quantification of flow cytometric apoptosis assay. **e** Western blot analysis of integrin β3, E-cadherin, and vimentin expression. **f** Cell viability assay in the presence or absence of 1 μM gefitinib. For all panels: *n* = 5. *** *P* < 0.001

To further establish a functional connection between miR-483-3p and integrin β3, we tested whether forced expression of integrin β3 can rescue the effect of miR-483-3p on gefitinib-resistant cells. Due to miR-483-3p targeting at 3′-UTR of ITGB3, we generated a lentiviral expression construct that encodes the entire ITGB3-coding sequence without its 3′-UTR and then transfected HCC827GR and HCC827 cells with this ITGB3-overexpression lentivirus (ITGB3). After integrin β3 was confirmed to be overexpressed and not inhibited by miR-483-3p (Fig. S3A), we overexpressed integrin β3 in miR-483-3p-expressing HCC827GR cells. In this rescue assay, overexpression of integrin β3 largely rescued miR-483-3p-mediated inhibitory growth effect (Fig. 6a upper lane and 6b), pro-apoptosis effect (Fig. 6d), EMT reversal (Fig. 6a middle lanes and 6e), invasion suppression (Fig. 6a lower lane and 6c), and gefitinib re-sensitization (Fig. 6f) in HCC827GR cells. These data demonstrated that integrin β3 was a key functional target, through which miR-483-3p exerted its effects on gefitinib-resistant NSCLC.

### miR-483-3p inactivates FAK/Erk signaling through repressing integrin β3

**Fig. 7:**
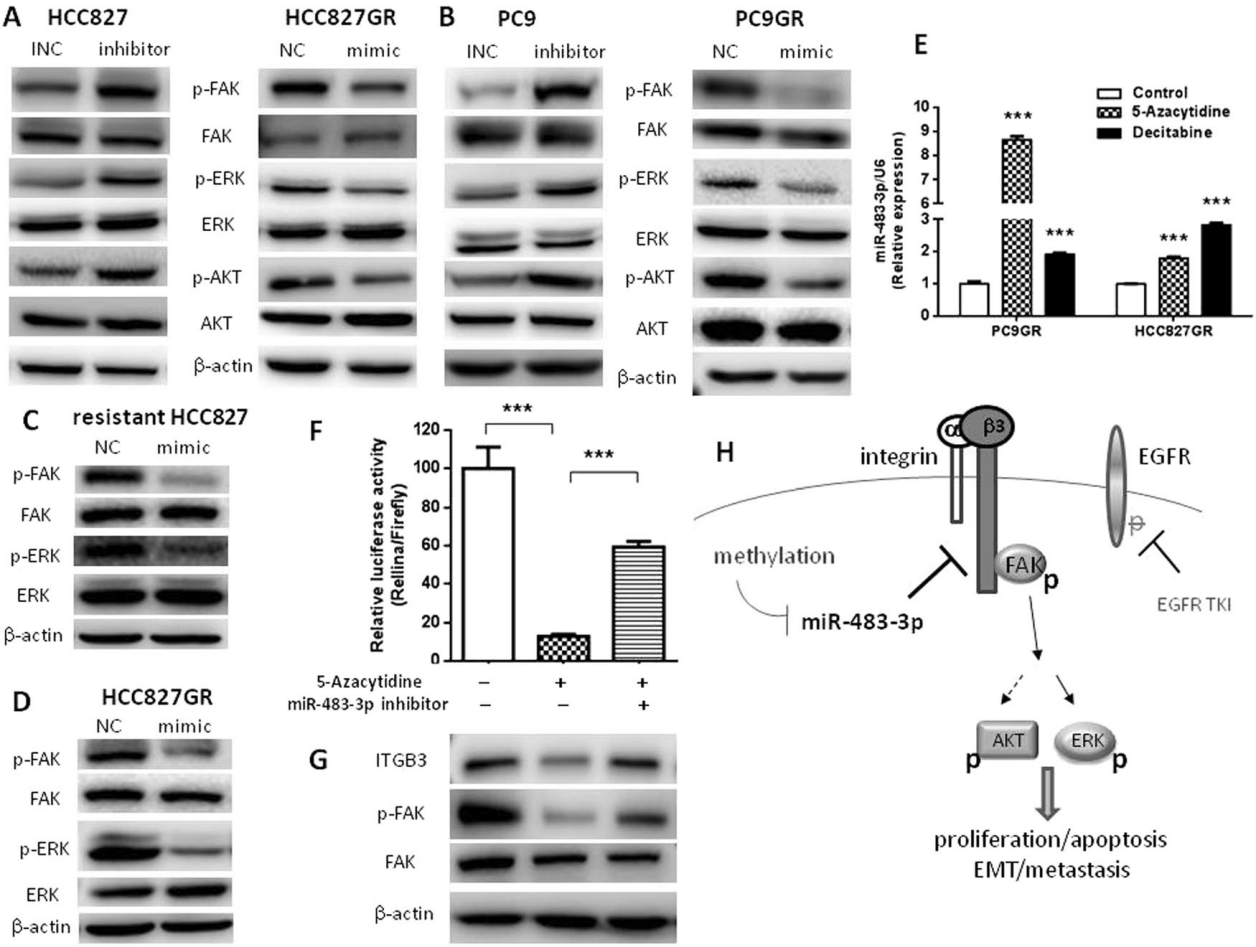
Downregulation of miR-483-3p activates FAK/Erk signaling and is due to hypermethylation of its promoter. **a**, **b** Western blot analysis of p-FAK, FAK, p-Erk, Erk, p-AKT, and AKT in indicated cells transiently transfected with miR-483-3p mimic (mimic), negative control (NC), miR-483-3p inhibitor (inhibitor), or inhibitor negative control (INC) as indicated. **c**, **d** Western blot analysis of p-FAK, FAK, p-Erk, and Erk in regrown/resistant HCC827 xenograft tumors (resistant HCC827) **c** and in HCC827GR-xenograft tumors **d** intratumorally injected with agomir-483-3p mimic (mimic) or agomir-NC (NC). **e** Quantitative RT-PCR analysis of miR-483-3p levels in indicated cells treated with 0.125 μM 5-azacytidine or 12.5 μg/ml decitabine for 72 h. **f** Luciferase activity in HCC827GR co-transfected with wild-type 3′-UTR of ITGB3 mRNA and miR-483-3p inhibitor (inhibitor) or inhibitor negative control (INC) in the presence or absence of 0.125 μM 5-azacytidine for 72 h as indicated. For all panels: *n* = 5. *** *P* < 0.001. **g** Western blot analysis of integrin β3, p-FAK, and FAK in HCC827GR cells transfected with miR-483-3p inhibitor (inhibitor) or inhibitor negative control (INC) in the presence or absence of 0.125 μM 5-azacytidine for 72 h as indicated. **h** Schematic diagram depicting the roles of miR-483-3p in acquired EGFR TKI resistance in NSCLC by targeting integrin β3

Integrins play important roles in many biological processes, including drug resistance and metastasis [34]. Given that FAK is a key component of the signaling pathways triggered by integrin [35], we next examined whether miR-483-3p regulates FAK and downstream signaling. Transient transfection of miR-483-3p mimic or inhibitor showed that miR-483-3p mimic markedly decreased the levels of FAK, Akt, and Erk phosphorylation in GR cells, whereas miR-483-3p inhibitor dramatically increased FAK, Akt, and Erk phosphorylation in parental cells (Fig. 7a, b). Moreover, integrin β3 overexpression largely rescued miR-483-3p-mediated downregulation of p-FAK, p-Akt, and p-Erk (Fig. S3B). To extend our finding to in vivo, we examined the phosphorylation of FAK, Akt, and Erk in our in vivo models. We found that miR-483-3p mimic treatment decreased FAK and Erk phosphorylation in both gefitinib-resistant HCC827-derived xenograft tumors (Fig. 7c and S3D) and HCC827GR-derived xenograft tumors (Fig. 7d and S3D) compared with NC treatment. Surprisingly, Akt phosphorylation was not changed by miR-483-3p treatment in either in vivo model (Fig.S3C and S3D). Collectively, our data demonstrated that miR-483-3p inactivated FAK/Erk signaling through repressing integrin β3 in vitro and in vivo.

### miR-483-3p is downregulated by promoter hypermethylation

At last, we explored the mechanisms underlying the silencing of miR-483-3p in gefitinib-resistant NSCLC. Epigenetic alterations, such as promoter DNA hypermethylation, can result in silencing of miRNA expression. Indeed, treatment with DNA-demethylating agents, 5-azacytidine or decitabine significantly restored miR-483-3p expression in both GR cell lines (Fig. 7e). 5-Azacytidine treatment reduced the expression levels of integrin β3 and FAK phosphorylation simultaneously and also decreased luciferase activity driven by wild-type 3′-UTR of ITGB3 mRNA in HCC827GR cells (Fig. 7f, g). Moreover, those effects of 5-azacytidine were largely rescued by miR-483-3p knockdown using miR-483-3p inhibitor in HCC827GR cells (Fig. 7f, g). Collectively, these data suggested that downregulation of miR-483-3p in gefitinib-resistant NSCLC is due to hypermethylation of its own promoter (Fig. 7h).

## Discussion

Acquired EGFR TKI resistance limits the long-term clinical efficacy of these drugs. Therefore, a better and more complete understanding of the mechanisms leading to acquired EGFR TKI resistance will be critical to develop treatment strategies. Recent evidence has demonstrated that miRNAs are important modulators of acquired EGFR TKI resistance [11, 12, 36]. In this study, we have first reported that dramatically decreased expression of miR-483-3p, which results from hypermethylation of its promoter, is functionally associated with acquired gefitinib resistance in cell lines and animal models of NSCLC. Furthermore, we have first identified integrin β3 as a functional target of miR-483-3p and a mediator of miR-483-3p-induced regulation of EGFR TKI-resistant NSCLC. However, it is also likely that miR-483-3p could target other unidentified proteins, which needs further study. More importantly, administration of miR-483-3p has significant efficacy in suppressing the growth of gefitinib-resistant tumors as well as re-sensitizing gefitinib-resistant tumors to gefitinib in vitro and in vivo, suggesting miR-483-3p as a potential therapeutic target for advanced lung cancer patients.

Hsa-miR-483 is a microRNA located within intron 2 of the human IGF2 locus. It has been reported that mature miR-483-3p is upregulated in some types of tumors [14–19], but downexpressed in others [20–23]. Functional studies have reported that miR-483-3p may act as an oncogene by targeting BBC3/PUMA directly [14] or tumor suppressor via repression of multiple targets including CDC25A, BIRC5, and RAN [37]. These discrepancies would be explained that miR-483-3p has opposite functions depending on its cellular context. It is not unusual of a miRNA that exerts distinct function in cancer depending on tumor type, tumor state and/or genetic background, such as miR-31 [38, 39]. In order to understand the roles of miR-483-3p in the tumorigenesis of lung adenocarcinoma, we examined the miR-483-3p level in lung adenocarcinoma compared with matching adjacent non-tumor tissues using multiple miRNA expression profiling data sets from TCGA and GEO database (Fig. S4A–S4C). The results revealed no significant change in miR-483-3p expression. Our qRT-PCR analysis also showed no significant alteration in miR-483-3p expression in six tested lung adenocarcinoma cell lines compared with two normal human lung epithelial cell lines (data not shown). Overexpression or knockdown of miR-483-3p in two immortalized normal human lung epithelial cell lines (BEAS2B and 16HBE) has no effect on their growth in vitro (Fig. S4D and S4E). Collectively, miR-483-3p may not participate in the tumorigenesis of lung adenocarcinoma, which needs further investigation.

EMT is closely related with treatment resistance and metastasis [33]. The acquisition of EMT phenotype has been detected in both NSCLC tumors and NSCLC cell lines with acquired EGFR TKI resistance [3, 6, 7, 24, 31, 32]. Consistently, EMT phenotype was observed in both gefitinib-resistant sublines and tumors in our study. Targeting EMT has been considered a promising strategy against drug resistance. Recently, miRNA has been reported to regulate EMT, thus representing attractive candidates for overcoming drug resistance, such as miR-200 family [40]. In our study, we found that forced expression of miR-483-3p reversed EMT in gefitinib-resistant NSCLC in vitro and in vivo, which contributes to the increased sensitivity to gefitinib. In addition, miR-483-3p overexpressing GR cells showed decreased migration and invasion in vitro and decreased metastasis in vivo, properties associated with EMT reversal. Taken together, miR-483-3p has great potential as a therapeutic target for advanced lung cancer patients by reversing EMT phenotype acquired during therapy.

To better comprehend the biological function of miR-483-3p mechanistically, we first identified integrin β3 as one of direct and functional targets for miR-483-3p based on several lines of evidence. First, miR-483-3p directly bound to seed-complementary binding sites in the 3′-UTR of integrin β3 mRNA and repressed upstream luciferase activity. Point mutations of several nucleotides in binding sites were adequate to abolish the effect of miR-483-3p. Second, the expressions of miR-483-3p and integrin β3 were inversely correlated in NSCLC cell lines. Third, up- and downregulation of miR-483-3p decreased and increased integrin β3, respectively. Last and more importantly, integrin β3 overexpression largely rescued the biological function mediated by forced expression of miR-483-3p. Integrins have been found to be required for tumor progression, metastasis, and treatment resistance [30, 34, 41]. Seguin and colleagues [30] reported that integrin β3 was upregulated after erlotinib treatment, which was consistent with our findings. More importantly, our findings add to current knowledge about how integrin β3 is upregulated in resistant tumors. According to their study, Kras/RalB/NF-κB pathway was essential for integrin β3-mediated erlotinib resistance. However, our results showed that upregulated integrin β3 in gefitinib-resistant cells resulting from miR-483-3p downregulation activated FAK/Erk or FAK/Akt/Erk pathway (Fig. 7h), which plays a critical role in invasion, metastasis, and EMT [42]. This discrepancy could be due to different cellular context and different treatment regimens. For example, Kanda and colleagues found that acquired erlotinib resistance was mediated by integrin β1/Src/Akt signaling pathway in lung cancer [41]. Consequently, additional investigations are needed to better understand the roles of miR-483-3p/integrin β3 signaling pathway in acquired resistance to EGFR TKI.

It is well established that promoter methylation is one of the most common mechanisms for aberrant expression of miRNA in human cancers. In this study, we provided several lines of evidence that promoter hypermethylation is a key modulator of miR-483-3p expression. First, in GR cells, 5-azacytidine or decitabine treatment significantly restored the miR-483-3p expression but reduced integrin β3 expression simultaneously. Second, 5-azacytidine treatment also decreased luciferase activity in GR cells transfected with ITGB3 3′-UTR wild-type. Last, miR-483-3p knockdown rescued the effects of 5-azacytidine treatment. Therefore, promoter hypermethylation seems to be used to downregulate miR-483-3p expression by gefitinib-resistant lung cancer cells. It is noteworthy that 5-azacytidine has been studied extensively and shown promising antitumor activity in vivo and is being evaluated for its potential clinical significance [43, 44]. Therefore, in addition to miR-483-3p replacement, demethylation of miR-483-3p promoter by 5-azacytidine is another approach that could prove effective in combination with EGFR TKI in advanced NSCLC treatment.

In summary, our study reported, for the first time, decreased expression of miR-483-3p caused by its promoter hypermethylation is functionally associated with acquired gefitinib resistance and acquisition of EMT in NSCLC. Functional and mechanistic studies suggest a mechanism by which downregulation of miR-483-3p activates FAK/Erk pathway through directly upregulating integrin β3 expression (Fig. 7h). More importantly, re-expression of miR-483-3p restored sensitivity to gefitinib, reversed EMT and inhibited migration, invasion, and metastasis in both in vitro and in vivo NSCLC models with acquired resistance to gefitinib. Our study provides new insight into mechanisms of acquired resistance to EGFR TKI and a potential target for combination therapy for lung patients with EGFR mutation.

## Materials and Methods

### Cell culture and establishment of NSCLC cell lines with acquired resistance to EGFR TKI in vitro

Human NSCLC HCC827, H1975, A549, H292, H1299 cells were obtained from the Cell Bank of Type Culture Collection of the Chinese Academy of Sciences and PC9 cells were kindly provided by Dr. Qianggang Dong in Shanghai Cancer Institute, Shanghai Jiao Tong University School of Medicine. All cell lines were examined by certified laboratories for authenticity using short tandem repeat analysis. Gefitinib- or afatinib-resistant cells were established as previously described [24]. Resistant cell lines are capable of proliferating normally in the present of 5 μM gefitinib (HY-50895, MedChemExpress, China) or afatinib (HY-10261 MedChemExpress, China). Cell viability was used to verify resistance after culturing cells in gefitinib- or afatinib-free medium for 5–7 days. Upon confirmation of resistance, resistant cell lines were growing in standard medium without gefitinib or afatinib and resistance to gefitinib or afatinib was tested regularly.

### Transfection of miRNA and lentiviral transduction

For transient transfection, cells were transfected with 100 nM mimic NC, miR-483-3p mimic, INC, or miR-483-3p inhibitor, (GenePharma, Shanghai, China) using Lipofectamine 3000 according to manufacturer’s instructions. The efficacy of transfection was verified by qRT-PCR.

For stable transfection, cells were transfected with pGCMV/EGFP/Blasticidin plasmids containing miR-483-3p mimic (shmimic) or negative control (shNC) (GenePharma). Stable cell clones were selected by blasticidin (Invitrogen) and then verified by qRT-PCR.

For integrin β3 rescue experiment, cells were infected with lentivirus containing the full-length human ITGB3 cDNA sequence (ITGB3) to overexpress integrin β3 (GeneCopoeia, Guanzhou, China) or GFP cDNA (LV) as a control.

### Reporter constructs and dual-luciferase assay

A total 883-bp nucleotide sequences corresponding to the 3′-UTR of ITGB3 (NM_000212) including the two predicted binding sites for miR-483-3p was cloned downstream of Rluc sequence in psiCHECK-2 vector (Promega) and verified by sequencing. QuikChange (Stratagene) site-directed mutagenesis was used to perform mutagenesis and mutations were also verified by sequencing. Dual-Luciferase Reporter Assay System (Promega) was used to measure luciferase activity. The sequences of primer are provided in the Supplementary Table S2.

### Mouse xenograft and metastasis models

Male athymic nude mice (BALB/c, 4–6 weeks old) were used for all the animal studies. All experimental procedures were approved by Shanghai Jiao Tong University.

To establish mouse xenograft models, same amount of indicated tumor cells was injected subcutaneously into both flanks of each mouse. The tumor volume was measured after 1 week from injection and then every other day or twice a week. Tumors were measured by investigators who were blinded to treatment plans. Tumor volumes (mm^3^) were calculated as length × width^2^/2. Mice were randomly distributed into groups, so that each group contained a similar group mean and median tumor volume (*n* = 5). In all studies, mice body weight was measured twice a week. All animals were included in the analysis.

To establish gefitinib-resistance NSCLC in vivo, gefitinib was given by gavage to HCC827-bearing mice at 25 mg/kg daily until sacrifice.

For miR-483-3p treatment study, 1 nmol agomir-483-3p mimic or agomir-NC (GenePharma) were intratumorally injected once every 4 days for a total four injections into left or right tumor of each mouse, respectively. After sacrifice, tumor nodules were dissected and weighted and then cut into two pieces with a scalpel. One was snap-frozen for subsequent Western blot and qRT-PCR analysis, whereas the other was fixed in fresh paraformaldehyde (4%) and embedded in paraffin for histology and immunohistochemistry analysis.

To establish mouse metastasis models, same amount of indicated tumor cells stably transfected with a luciferase reported gene was injected intravenously via lateral tail veins into each mouse. The whole body metastasis was examined using bioluminescence imaging.

### Statistical analysis

All data are presented as the mean±SEM. Sample sizes in animal experiments were selected on the basis of similar experiments in literature. Statistical analyses were performed using a two-tailed *t*-test (GraphPad Prism 7.0), except that the variance was unequal between two groups. In such cases, a Welch’s corrected *t*-test was applied. Correlations between miR-483-3p and ITGB3 expressions were examined using two-tailed Pearson’s rank correlation coefficient. *P* values of < 0.05 were considered statistically significant. Experiments were all repeated at least three times in vitro and twice in vivo.

## Electronic supplementary material

Supplementary Methods and Material

Supplementary Figure legends

Supplementary Figure S1

Supplementary Figure S2

Supplementary Figure S3

Supplementary Figure S4

Supplementary Figure S5

Supplementary Tables
