# Supplementary material for: Epigenetic silencing of miR-483-3p promotes acquired gefitinib resistance and EMT in EGFR-mutant NSCLC by targeting integrin β3
Source: Oncogene. 2018 May 2;37(31):4300–12. doi: 10.1038/s41388-018-0276-2 (PMC6072709; doi:10.1038/s41388-018-0276-2)
Supplement: Supplementary file 1 — Supplementary Methods and Material [file 41388_2018_276_MOESM1_ESM.docx]

**SuPPLEMENTARY Materials and Methods**

**RNA isolation and qRT-PCR**

RNA was extracted according to manufacturer's instructions using miRNA Isolation Kit (Invitrogen). qRT-PCR analysis for miR-483-3p was performed with [TaqMan MicroRNA Reverse Transcription Kit](https://www.thermofisher.com/order/catalog/product/4366596?ICID=search-product) (Thermo Fisher catalog#[4366596](https://www.thermofisher.com/order/catalog/product/4366596)) and TaqMan MicroRNA Assay (Thermo Fisher catalog# 4427975). Relative miRNA expression was calculated by the comparative Ct method using RNU6B for normalization. The primer sequences for RNU6B are as following: Forward 5’ TCGCTTCGGCAGCACATA 3’; Reverse 5’ TTCACGAATTTGCGTGTCAT 3’.

**miRNA microarray**

Microarray experiments were performed by the National Engineering Center for Biochip at Shanghai (Shanghai Biotechnology Corporation) following standard Agilent protocols. Briefly, total RNAs were prepared from cells using mirVana miRNA isolation kit (Ambion), and then labeled and hybridized using miRNA Complete Labeling and Hyb Kit (Agilent). Arrays were scanned on an Agilent Microarray Scanner and the data were extracted using Agilent Feature Extraction Software. After background subtraction, the data were imported into Gene Spring software (Agilent) for further analysis. miRNAs with expression levels differing by at least two fold between both parent cell lines and their respective gefitinib-resistant cell lines were selected. The data were deposited into Gene Expression Omnibus (GSE110815).

**Cell viability assay**

Cells were cultured in the presence of drugs or vehicle for 72 hours, and viability was determined using the Cell Counting Kit-8 (CCK8) colorimetric assay (Dojindo, Shanghai, China) according to the manufacturer’s instructions.

**Proliferation assay**

5-Ethynyl-2'-deoxyuridine (EdU) incorporation assay was used to directly detect proliferation by immunofluorescence of newly synthesized DNA. Briefly, cells were incubated with EdU for 2-4 hours and then processed according to manufacturer's instructions using Cell-Light EdU Apollo 643 In Vitro Imaging Kit (Ribobio, Guanzhou, China). Proliferation was determined as percentage of EdU-positive cells among Hoechst-stained cells.

**Apoptosis assay**

Apoptosis was assessed using Annexin V staining kit according to the manufacturer's instructions (Dojindo). Briefly, cells were collected and incubated with Annexin V and propidium iodide for 30 minutes and then analyzed using flow cytometry.

**Western blot, flow cytometry and immunofluorescence**

Protein expression was measured with Western blot, flow cytometry or immunofluorescence as previously described [24]. β-actin was used as loading control for Western blot. A list of antibodies used is available in the Supplementary Table S1.

**Proliferation assay**

A real-time imaging system (IncuCyte) was also used to measure cell proliferation using non-label cell monolayer confluence approach. Briefly, 5000 cells were seeded into 96-well plates containing DMEM supplemented with 10% FBS. The plates were placed into an IncuCyte Zoom (Essen Bioscience) that automatically takes phase-contrast images in each well every 2 hours over the course of 2-5 days and utilizes software to measure confluence as a proxy for proliferation.

**Colony formation assay**

For clonogenicity analysis, 72 hours after transfection, 800-1000 viable cells were placed in six-well plates and cultured in complete medium for 2-3 weeks. Colonies were fixed, stained with crystal violet and then counted.

**5-Azacytidine or decitabine treatment**

Cells were treated with 5-azacytidine (0.125μM) or decitabine (12.5μg/ml) for 72 hours with a change of culture medium every 24 hours.
