# Supplementary material for: Epigenetic silencing of miR-483-3p promotes acquired gefitinib resistance and EMT in EGFR-mutant NSCLC by targeting integrin β3
Source: Oncogene. 2018 May 2;37(31):4300–12. doi: 10.1038/s41388-018-0276-2 (PMC6072709; doi:10.1038/s41388-018-0276-2)
Supplement: Supplementary file 2 — Supplementary Figure legends [file 41388_2018_276_MOESM2_ESM.docx]

**Supplementary Figure legends**

**Figure S1. The expression of miR-483-3p is dramatically downregulated in EGFR TKI-resistant NSCLC cell lines**

(A) miRNA microarray analysis of PC9GR and HCC827GR compared with their parent cell lines PC9 and HCC827, respectively. (GSE110815) (B) Quantitative RT-PCR analysis of miR-483-3p levels in indicated cell lines. RNU6B is used for normalization. AR: afatinib-resistant. For all panels: n=5. *** P< 0.001.

**Figure S2. Efficacy of miR-483-3p mimic and inhibitor**

(A) Quantitative RT-PCR analysis of miR-483-3p levels in HCC827GR and PC9GR transiently transfected with miR-483-3p mimic (mimic) or negative control (NC). (B) Quantitative RT-PCR analysis of miR-483-3p levels in HCC827 and PC9 transiently transfected with miR-483-3p inhibitor (inhibitor) or inhibitor negative control (INC). (C,D) Quantitative RT-PCR analysis of miR-483-3p levels in regrown/resistant HCC827 xenograft tumors (C) or HCC827GR xenograft tumors (D) intratumorally injected with 1 nmol agomir-483-3p mimic (mimic) or agomir-NC (NC) once every four days for a total four injections.(E) Quantitative RT-PCR analysis of miR-483-3p levels in HCC827GR stably transfected with miR-483-3p mimic (shmimic) or negative control (shNC). RNU6B is used for normalization. For all panels: n=5. **P<0.01; *** P< 0.001.

**Figure S3. miR-483-3p regulates cell proliferation**

Quantification of EdU incorporation assays of Figure 2A (A) and Figure 2C (B). For all panels: n=5. **P<0.01.

**Figure S4. miR-483-3p inactivates FAK/Erk signaling by suppressing integrin β3 expression**

(A) Western blot analysis of integrin β3 of HCC827GR and HCC827 co-transfected with miR-483-3p mimic (mimic) and LV-ITGB3 (ITGB3) or LV-GPF (LV) as indicated. (B) Western blot analysis of p-FAK, FAK, p-Erk, Erk, p-AKT and AKT in HCC827GR co-transfected with miR-483-3p mimic and/or LV-ITGB3 (ITGB3) as indicated. (C) Western blot analysis of p-AKT and AKT of regrown/resistant HCC827 xenograft tumors (resistant) (left panel) and HCC827GR xenograft tumors (right panel) intratumorally injected with agomir-483-3p mimic (mimic) or agomir-NC (NC). (D) Immunostaining analysis of p-AKT and p-Erk in sectioned xenograft tumors of (C).

**Figure S5. miR-483-3p is not associated with tumorigenesis of lung adenocarcinoma**

(A-C) The expression levels of miR-483-3p in lung adenocarcinoma with paired adjacent non-tumor tissues in dataset GSE63805 (A), GSE36681 (B) and TCGA (C). (D, E) The growth curve of BEAS2B (D) and 16HBE (E) transiently transfected with miR-483-3p mimic (mimic), negative control (NC), miR-483-3p inhibitor (inhibitor) or inhibitor negative control (INC) or Lipofectamine 3000 (lipo) as indicated. Cell proliferation was measured by real-time imaging system (IncuCyte).
