# Supplementary figures and images for: Epigenetic silencing of miR-483-3p promotes acquired gefitinib resistance and EMT in EGFR-mutant NSCLC by targeting integrin β3
Source: Oncogene. 2018 May 2;37(31):4300–12. doi: 10.1038/s41388-018-0276-2 (PMC6072709; doi:10.1038/s41388-018-0276-2)

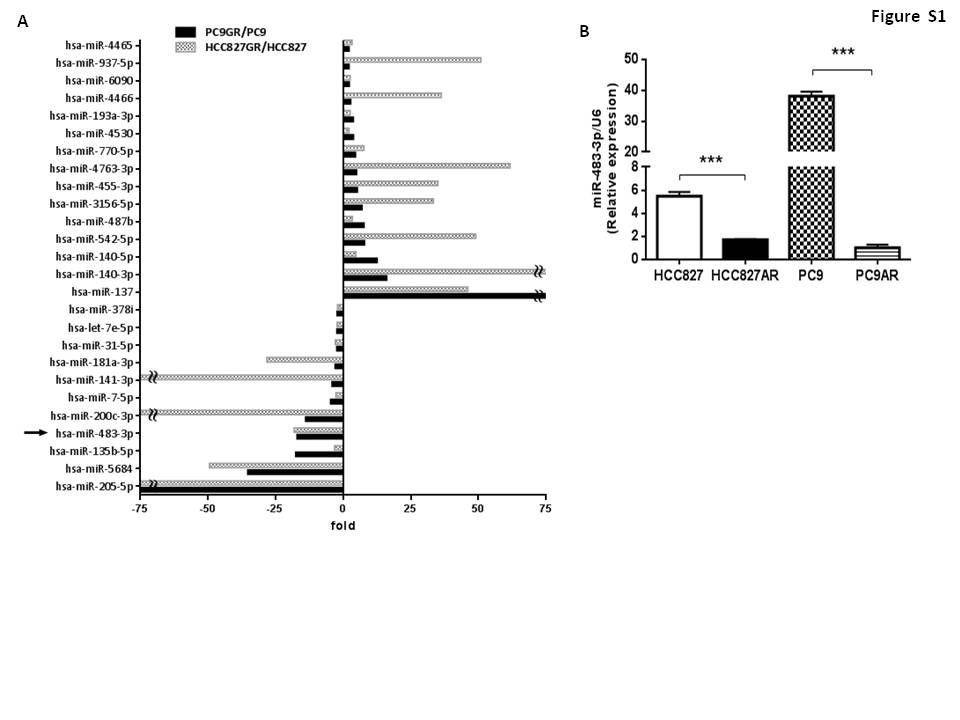

Supplement: Supplementary file 3 — Supplementary Figure S1 [file 41388_2018_276_MOESM3_ESM.jpg]

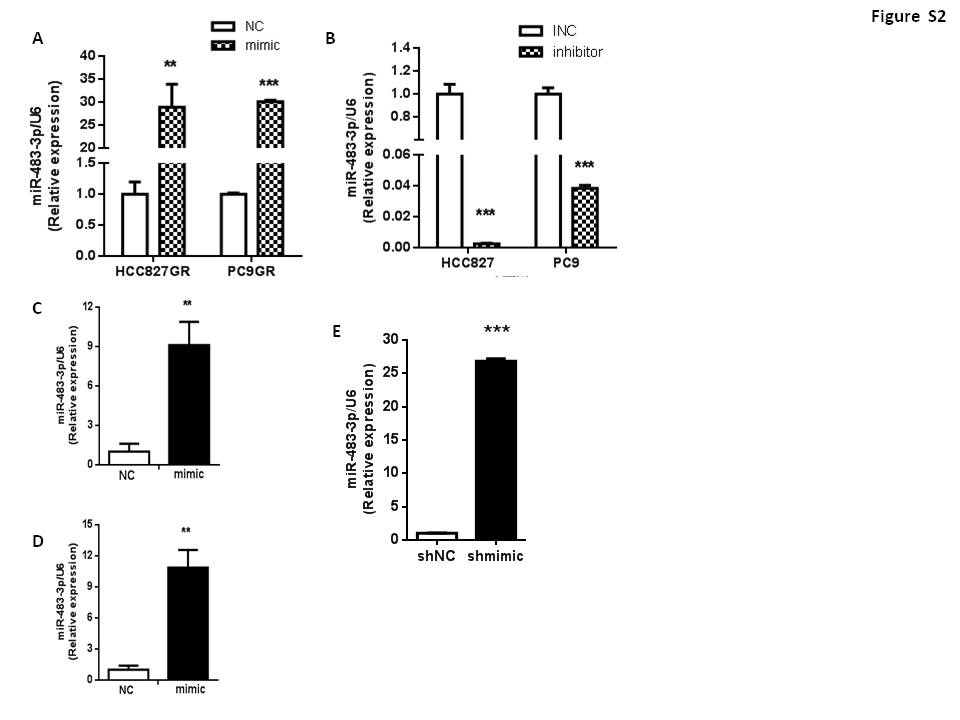

Supplement: Supplementary file 4 — Supplementary Figure S2 [file 41388_2018_276_MOESM4_ESM.jpg]

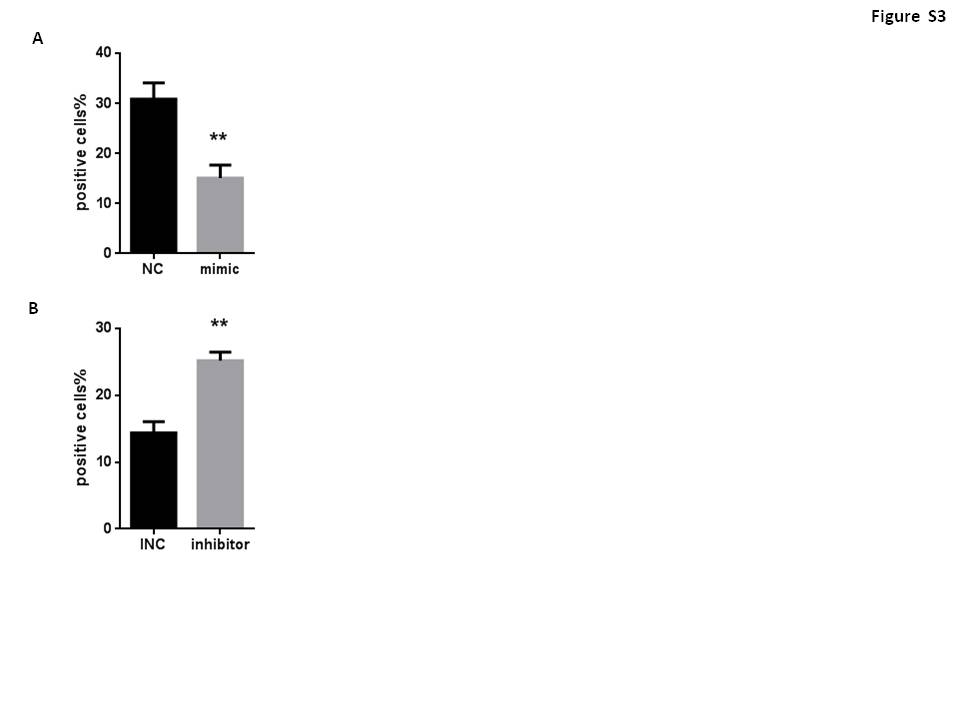

Supplement: Supplementary file 5 — Supplementary Figure S3 [file 41388_2018_276_MOESM5_ESM.jpg]

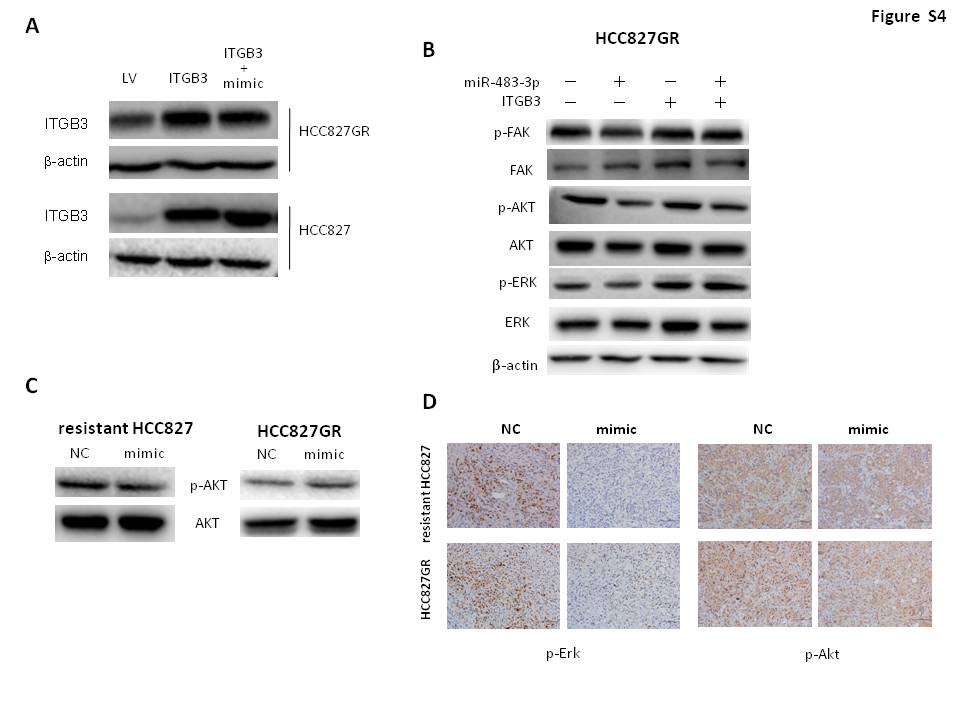

Supplement: Supplementary file 6 — Supplementary Figure S4 [file 41388_2018_276_MOESM6_ESM.jpg]

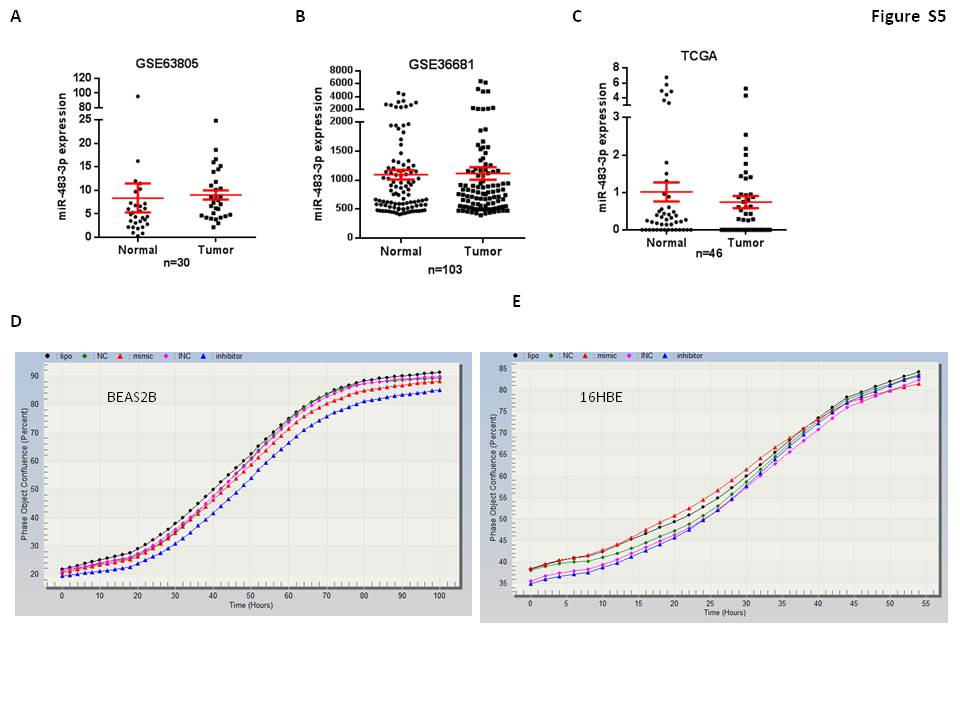

Supplement: Supplementary file 7 — Supplementary Figure S5 [file 41388_2018_276_MOESM7_ESM.jpg]
