# Supplementary material for: Epigenetic silencing of miR-483-3p promotes acquired gefitinib resistance and EMT in EGFR-mutant NSCLC by targeting integrin β3
Source: Oncogene. 2018 May 2;37(31):4300–12. doi: 10.1038/s41388-018-0276-2 (PMC6072709; doi:10.1038/s41388-018-0276-2)
Supplement: Supplementary file 8 — Supplementary Tables [file 41388_2018_276_MOESM8_ESM.docx]

**Supplementary TaBLEs**

| **Table S1. Antibodies** | | | |
| --- | --- | --- | --- |
| **Target** | **Use** | **Vendor** | **Catalog Number** |
| PARP | WB | Cell Signaling Tech | 9542 |
| Cleaved PARP | WB | Cell Signaling Tech | 5625 |
| Caspase-3 | WB | Cell Signaling Tech | 9665 |
| Cleaved Caspase-3 | WB | Cell Signaling Tech | 9664 |
| β-actin | WB | Santa Cruz Biotechnology | sc-47778 |
| E-cadherin | WB, IF | Cell Signaling Tech | 3195 |
| β -catenin | WB | Cell Signaling Tech | 8480 |
| Vimentin | WB, IF | Cell Signaling Tech | 5741 |
| Snail | WB | Cell Signaling Tech | 3879 |
| Zeb1 | WB | Cell Signaling Tech | 3396 |
| CD133 | WB | Biorbyt | orb99113 |
| CD44 | WB | Cell Signaling Tech | 3570 |
| Nanog | WB | Cell Signaling Tech | 4903 |
| Sox2 | WB | Cell Signaling Tech | 3579 |
| ITGB3 | WB, IHC | Cell Signaling Tech | 13166 |
| pFAK | WB | Cell Signaling Tech | 8556 |
| FAK | WB | Cell Signaling Tech | 3285 |
| pERK | WB, IHC | Cell Signaling Tech | 4376 |
| ERK | WB | Cell Signaling Tech | 4695 |
| pAKT | WB, IHC | Cell Signaling Tech | 4060 |
| AKT | WB | Cell Signaling Tech | 4691 |
| CD133 | flow cytometry | Miltenyl Biotec | 130–098–826 |
| CD44 | flow cytometry | BD Pharmingen | 555478 |

| **Table S2. Primers used for cloning and mutagenesis** | | |
| --- | --- | --- |
| **GENE** | **Forward Primer** | **Reverse Primer** |
| 3’UTR ITGB3 WT | GAGTCTCGAGATAAGCAGTCATCCTCAGATCATTATCAG | CTCGCGGCCGCCATAGCAAATGTCTACAGCAGTGAGGGTGTG |
| 3’UTR ITGB3 M1 | GTGTTTATGTGTGTGTGTTGTGTGTGCCTCAGTGTAATTTAAAATTGTGATGTGTCCTG | CAGGACACATCACAATTTTAAATTACACTGAGGCACACACAACACACACACATAAACAC |
| 3’UTR ITGB3 M2 | CTTGCAAGCTAATTCTTTGACCTGTTGGCCTCACGGATGTCTGGGCCACTCAGGGGTC | GACCCCTGAGTGGCCCAGACATCCGTGAGGCCAACAGGTCAAAGAATTAGCTTGCAAG |
